# Re-exploration of U’s Triangle *Brassica* Species Based on Chloroplast Genomes and 45S nrDNA Sequences

**DOI:** 10.1038/s41598-018-25585-4

**Published:** 2018-05-09

**Authors:** Chang-Kug Kim, Young-Joo Seol, Sampath Perumal, Jonghoon Lee, Nomar Espinosa Waminal, Murukarthick Jayakodi, Sang-Choon Lee, Seungwoo Jin, Beom-Soon Choi, Yeisoo Yu, Ho-Cheol Ko, Ji-Weon Choi, Kyoung-Yul Ryu, Seong-Han Sohn, Isobel Parkin, Tae-Jin Yang

**Affiliations:** 1 Genomics Division, National Institute of Agricultural Sciences, Jeonju, 54874 Korea; 2 International Technology Cooperation Center, Rural Development Administration, Jeonju, 54875 Korea; 3 Department of Plant Science, Plant Genomics and Breeding Institute, Research Institute of Agriculture and Life Sciences, College of Agriculture and Life Sciences, Seoul National University, Seoul, 08826 Republic of Korea; 4 Agriculture and Agri-Food Canada, 107 Science Place, Saskatoon, SK S7N 0X2 Canada; 5 Joeun Seed, Goesan-Gun, Chungcheongbuk-Do 28051 Republic of Korea; 6 Phyzen Genomics Institute, Seongnam, 13558 Republic of Korea; 7 National Agrobiodiversity Center, National Institute of Agricultural Sciences, RDA, Jeonju, Jeollabukdo 54874 Republic of Korea; 8 Postharvest Technology Division, National Institute of Horticultural and Herbal Science, Wanju, Republic of Korea; 9 Crop Biotechnology Institute/GreenBio Science and Technology, Seoul National University, Pyeongchang, 232-916 Republic of Korea

## Abstract

The concept of U’s triangle, which revealed the importance of polyploidization in plant genome evolution, described natural allopolyploidization events in *Brassica* using three diploids [*B*. *rapa* (A genome), *B*. *nigra* (B), and *B*. *oleracea* (C)] and derived allotetraploids [*B*. *juncea* (AB genome), *B*. *napus* (AC), and *B*. *carinata* (BC)]. However, comprehensive understanding of *Brassica* genome evolution has not been fully achieved. Here, we performed low-coverage (2–6×) whole-genome sequencing of 28 accessions of *Brassica* as well as of *Raphanus sativus* [R genome] to explore the evolution of six *Brassica* species based on chloroplast genome and ribosomal DNA variations. Our phylogenomic analyses led to two main conclusions. (1) Intra-species-level chloroplast genome variations are low in the three allotetraploids (2~7 SNPs), but rich and variable in each diploid species (7~193 SNPs). (2) Three allotetraploids maintain two 45SnrDNA types derived from both ancestral species with maternal dominance. Furthermore, this study sheds light on the maternal origin of the AC chloroplast genome. Overall, this study clarifies the genetic relationships of U’s triangle species based on a comprehensive genomics approach and provides important genomic resources for correlative and evolutionary studies.

## Introduction

Brassicaceae is one of the largest eudicot families; it contains more than 330 genera and 3,800 species. The genomes of species in the tribe Brassiceae share a common whole-genome triplication, which is considered to be a crucial event that drove diversification of the species and intra-species morphotypes^1,2^. Brassiceae includes several economically important crops that are used for vegetables, oils, and fodders. The basic foundation for the systematic relationship of the six major *Brassica* species was classically explained as U’s triangle^3^. U’s triangle proposed that the three tetraploid species *B*. *juncea* (AABB genome, 2*n* = 4*x* = 36), *B*. *napus* (AACC, 2*n* = 4*x* = 38), and *B*. *carinata* (BBCC, 2*n* = 4*x* = 34) are the derived allotetraploids of the diploid species *B*. *rapa* (AA, 2*n* = 2*x* = 20), *B*. *nigra* (BB, 2*n* = 2*x* = 16), and *B*. *oleracea* (CC, 2*n* = 2*x* = 18), respectively, which arose by natural hybridization and chromosome doubling.

Whole-genome sequencing (WGS) analyses of the A, C, AB, and AC genomes has increased our understanding of *Brassica* genome evolution^4–8^. It has been suggested that the *Brassica* genome diverged from *Arabidopsis thaliana* around 17 million years ago (mya)^9^, and there is evidence that the B genome first diverged from the *Brassica* lineage around 9 mya, followed by divergence of the A and C genomes around 4.5 mya^10,11^. Recent genome sequencing of the two AC and AB genome allotetraploids suggested that they derive from allotetraploidization events that happened approximately 8,000~51,000 years ago^6,8^.

Cells contain three different genomes (nuclear, mitochondrial, and chloroplast) that follow different evolutionary pathways^12^. Chloroplast, mitochondrial, and nuclear ribosomal DNA sequences are crucial resources to understand plant genomic diversity due to their highly conserved nature and strong phylogenetic signals. The chloroplast genome is circular, relatively simple, and inherited uniparentally with a highly conserved gene structure and gene order^13,14^. The chloroplast genome has sufficiently informative nucleotide divergence that it can be utilized to understand genetic diversity, genomic origin, and genetic relationships, as well as for barcode marker development^15–19^. A few systematic studies have explored the *Brassica* chloroplast genome; however, these results have yielded a partial and unresolved understanding^20–23^.

Nuclear ribosomal DNA (nrDNA) sequences are highly homozygous, tandemly repeated transcriptional units that encode important housekeeping functions in nuclear assembly and nuclear function^24,25^. Two nuclear ribosomal DNA blocks, 5SnrDNA and 45SnrDNA, are generally localized on different chromosomes in plants. The 45SnrDNA units contain a highly conserved multicistronic gene with 18S, 5.8S, and 28S RNA sequences and relatively polymorphic internal transcribed spacer (ITS) regions, which makes 45SnrDNA a preferred target for both phylogenetic and barcoding analyses^26–28^.

Advances in next-generation sequencing (NGS) technology and bioinformatics algorithms are facilitating the discovery of extensive natural variations in large populations. Most research has focused on identification of intra-species natural variations in the nuclear genome to explore diversity, adaptation, domestication, and evolution, as well as to mine for new alleles^29^. Our group recently established a method based on ‘genome skimming’ approach called dnaLCW for high-throughput simultaneous *de novo* assembly of chloroplast and 45SnrDNA transcription unit sequences using low-coverage whole-genome NGS to reveal inter-species and intra-species diversity^30–32^.

The objective of the current study is to elucidate the genetic diversity and evolution of *Brassica* species belonging to U’s triangle by performing whole-genome sequencing (WGS). We report the complete sequences of chloroplast genomes and 45SnrDNA transcription units for 28 genotypes. We also investigate genome-wide variation and phylogenomic analysis for chloroplast genomes and 45SnrDNA sequences to revisit the evolution of the six *Brassica* species in U’s triangle compared with the related species *Raphanus sativus*.

## Results

### Characterization of 28 complete chloroplast genomes

**Figure 1:**
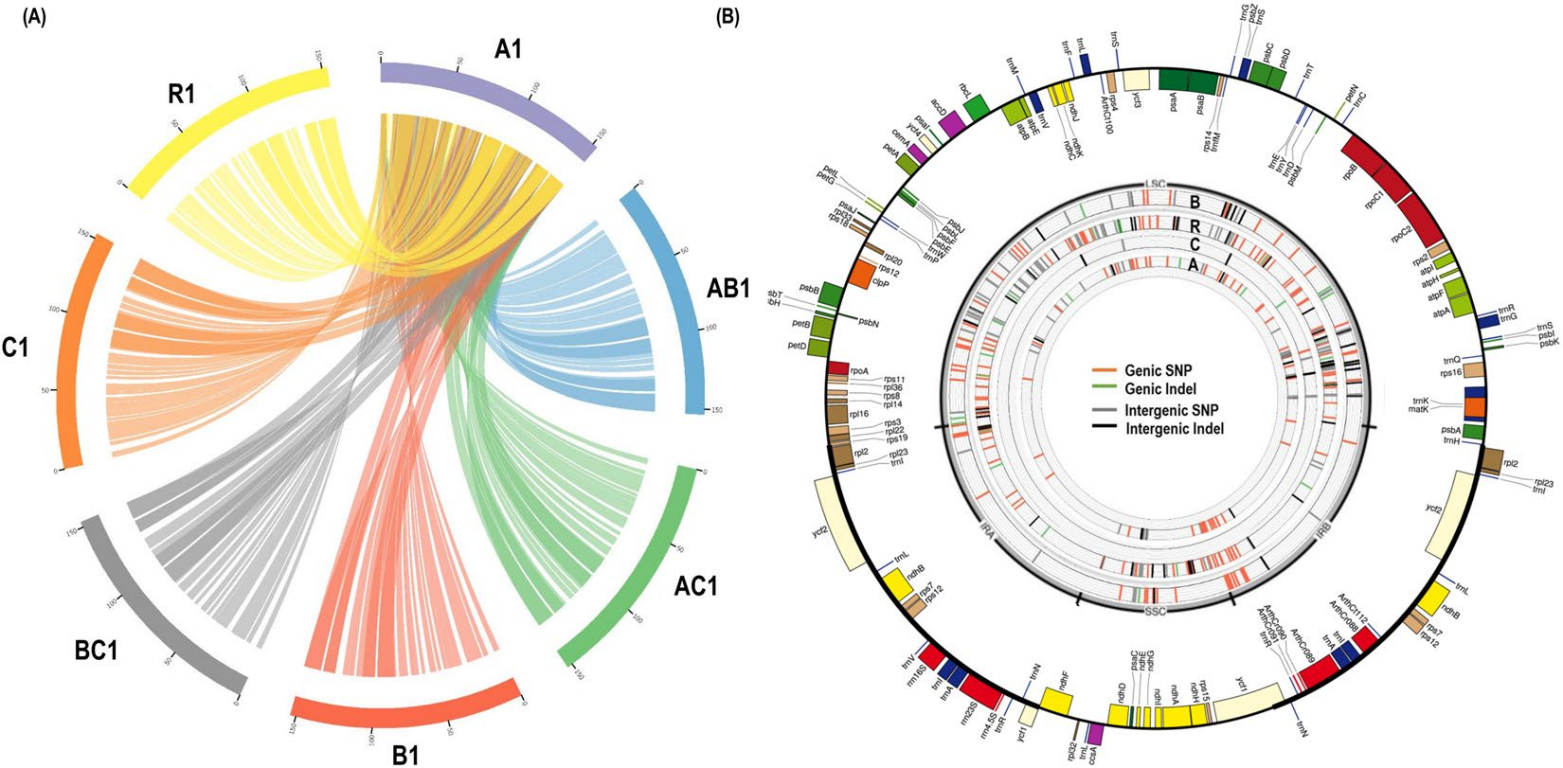
Chloroplast genome variations and comparative analysis in seven Brassicaceae species. (**A**) Synteny comparisons of chloroplast genomes in *Brassica*. Circos-based syntenic comparative map developed for *B*. *rapa* (A1) against *B*. *juncea* (AB1), *B*. *napus* (AC1), *B*. *nigra* (B1), *B*. *carinata* (BC1), *B*. *oleracea* (C1), and *Raphanus sativus* (R1). Syntenic blocks with minimum length of 1 kb were used for the syntenic analysis. (**B**) Distribution of intra-species variations in *B*. *nigra* (**B**), *R*. *sativus* (R), *B*. *oleracea* (**C**), and *B*. *rapa* (R) chloroplast genomes. Outermost chloroplast circular map was developed from the *B*. *rapa* chloroplast genome (A1) using OGDRAW. Genes are represented in different colors. Positive and negative gene orientations are shown as outer and inner circles, respectively. Inner circles represent variations in the B, R, C, and A genomes, respectively.

**Figure 2:**
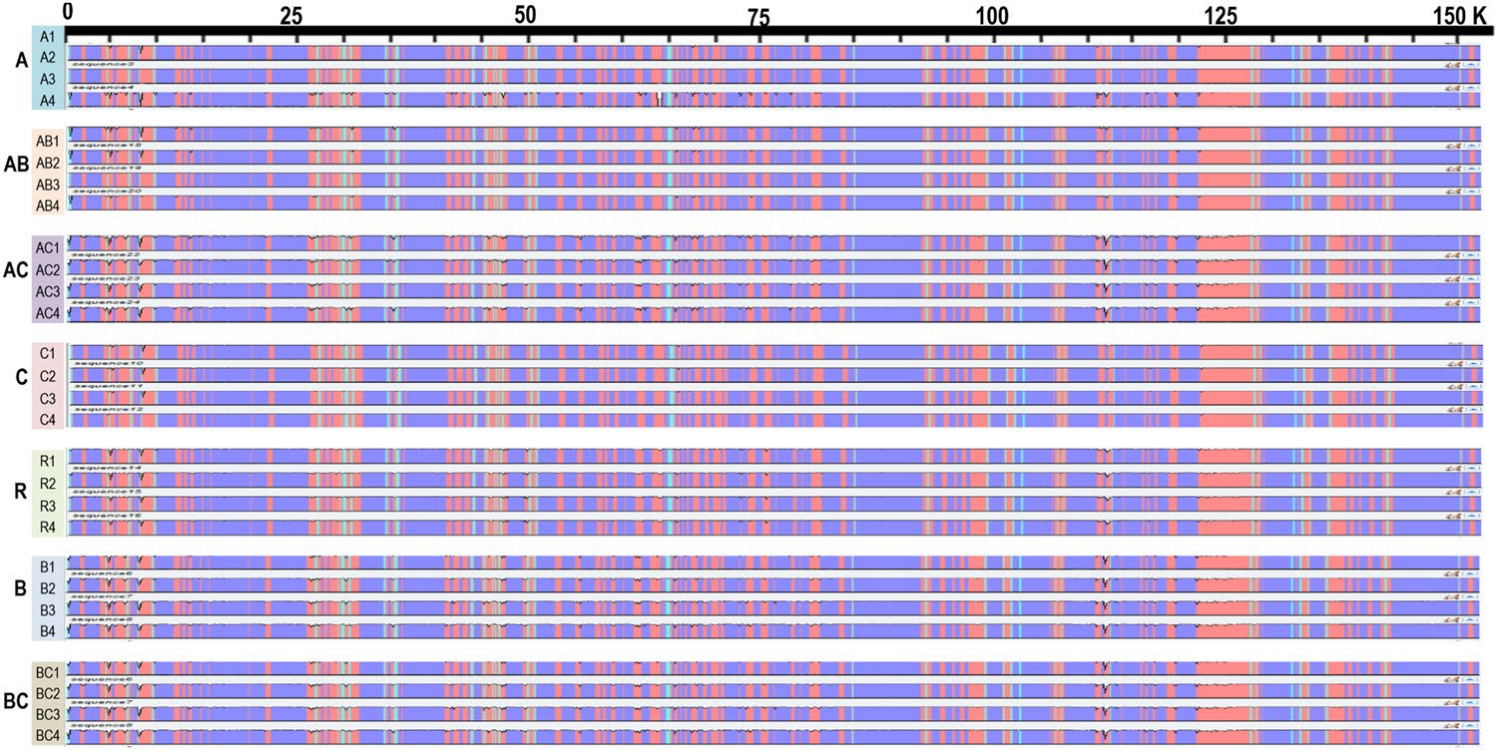
Comparative analysis based on complete chloroplast genomes identify similar and variable regions among the 28 *Brassica* and *Raphanus* genotypes.

The complete chloroplast genomes were obtained for 28 genotypes using the dnaLCW approach (Table 1). Annotation of chloroplast genomes revealed conserved quadripartite structures with coherent gene number and gene order among the 28 genotypes (Fig. 1). The chloroplast genome is highly conserved, with 99–100% sequence similarity within each species, although meaningful variations were observed between species with 98.1–99.5% sequence similarity (Fig. 2; Table S1). Chloroplast genome lengths varied by 607 bp among the 28 genotypes, ranging from 153,037 bp (accession A4) to 153,642 bp (accession B4). Chloroplast genome copy numbers were estimated based on read depth for the haploid genome size, ranging from 453 (accession AB2) to 1,279 (accession BC1) copies per cell (Table 1).

**Table 1:** Summary of chloroplast and 45SnrDNA assemblies from 28 *Brassica* and *Raphanus* genotypes.

Organism and genome	Genotype ID^a^	Genome size (Mb)	Total reads (Mb)	Chloroplast genome			45SnrDNA		
				Length (bp)	Copy number (x)^b^	Accession number	Length (bp)	Copy number (x)^b^	Accession number
*B*. *rapa* (A)	A1	529	1,557	153,483	378	KX681647	5,818	3,216	KX709342
	A2	529	1,214	153,482	305	KX681648	5,818	3,770	KX709343
	A3	529	1,352	153,482	363	KX681649	5,818	3,872	KX709344
	A4	529	1,293	153,037	496	KX681650	5,818	4,183	KX709345
*B*. *nigra* (B)	B1	632	1,532	153,633	378	KT878383	5,831	1,819	KX709346
	B2	632	1,632	153,641	221	KX681651	5,831	1,667	KX709347
	B3	632	1,489	153,623	323	KX681652	5,831	1,324	KX709348
	B4	632	1,631	153,642	244	KX681653	5,831	1,571	KX709349
*B*. *oleracea* (C)	C1	630	1,489	153,364	278	KX681654	5,811	2,873	KX709350
	C2	630	1,312	153,364	510	KX681655	5,848	1,384	KX709351
	C3	630	1,611	153,364	285	KX681656	5,818	2,768	KX709352
	C4	630	2,115	153,363	347	KX681657	5,819	1,957	KX709353
*R*. *sativus* (R)	R1	530	1,467	153,372	264	KX681658	5,816	3,812	KX709354
	R2	530	1,487	153,444	412	KX681659	5,816	2,042	KX709355
	R3	530	1,440	153,376	393	KX681660	5,819	4,174	KX709356
	R4	530	1,470	153,370	343	KX681661	5,823	4,614	KX709357
*B*. *juncea* (AB)	AB1-A	1,068	1,469	153,483	779	KX681662	5,818	2,412	KX709358
	AB1-B						5,831	1,589	KX709359
	AB2-A	1,068	1,352	153,483	358	KX681663	5,818	1,883	KX709360
	AB2-B						5,831	690	KX709361
	AB3-A	1,068	1,528	153,490	495	KX681664	5,818	2,192	KX709362
	AB3-B						5,831	1,041	KX709363
	AB4-A	1,068	1,549	153,483	338	KX681665	5,818	3,449	KX709364
	AB4-B						5,831	1,190	KX709365
*B*. *napus* (AC)	AC1-A	1,130	1,534	153,452	630	KX681666	5,831	1,445	KX709366
	AC1-C						5,818	689	KX709367
	AC2-A	1,130	1,401	153,429	890	KX681667	5,831	1,169	KX709368
	AC2-C						5,819	879	KX709369
	AC3-A	1,130	1,401	153,429	925	KX681668	5,817	1,009	KX709370
	AC3-C						5,832	865	KX709371
	AC4-A	1,130	1,579	153,453	366	KX681669	5,831	982	KX709372
	AC4-C						5,818	741	KX709373
*B*. *carinata* (BC)	BC1-B	1,284	2,156	153,636	762	KX681670	5,818	4,223	KX709374
	BC1-C						5,818	2,409	KX709375
	BC2-B	1,284	1,457	153,636	919	KX681671	5,818	5,865	KX709376
	BC2-C						5,818	3,453	KX709377
	BC3-B	1,284	1,710	153,641	913	KX681672	5,818	2,813	KX709378
	BC3-C						5,817	1,836	KX709379
	BC4-B	1,284	1,511	153,636	540	KX681673	5,818	4,791	KX709380
	BC4-C						5,818	2,551	KX709381

^a^rDNA from tetraploids was designated as A, B, or C based on the parental genome or sub-genome type. The complete details and list of organisms can be found in Table S6. ^b^Copy numbers of chloroplast and 45SnrDNA were estimated based on average read depth mapping and converted into the corresponding haploid genome size.

The chloroplast genomes showed different levels of intra-species polymorphism (Tables 2, S2, S3). The chloroplast genomes from C genome species had very low intra-species diversity with seven SNPs and four InDels, whereas other chloroplast genomes had relatively high intra-species diversity with 88 SNPs and 16 InDels in the A species genomes, 99 and 24 in the B species genomes, and 193 and 112 in the R genome, respectively (Tables S2, S3). Polymorphism was richer in genic regions than in intergenic regions (Table 3). Abundant polymorphisms were detected on the inter-species level (Tables 2, S4). The highest number of inter-species variations was 2,502 SNPs and 294 InDels between the B and C chloroplast genomes, and the lowest was 257 SNPs and 65 InDels between the A and C chloroplast genomes (Table S4). The three tetraploids showed fewer variations in the chloroplast genome compared to the diploid species.

**Table 2:** Summary of inter-species and intra-species variations based on chloroplast genomes.

SNP/Indel	A	B	C	R
A	88/16^a^	280^b^	65	167
B	2,402^b^	99/24	294	245
C	257	2,502	7/4	183
R	1,203	2,259	1,293	193/112

^a^18/16 denotes the number of SNP/Indel variations in the A genome. ^b^B genome has 2,402 and 280 SNP and InDel variations, respectively, compared with the A genome.

**Table 3:** Summary and distribution of intraspecies SNP and Indel variations based on chloroplast genomes.

Genome	SNP			Indel		
	Genic	Intergenic	Total	Genic	Intergenic	Total
A	58	30	88	8	8	16
B	68	31	99	13	11	24
C	2	5	7	3	1	4
R	137	56	193	57	55	112

### Characterization of 45SnrDNA sequences

**Figure 3:**
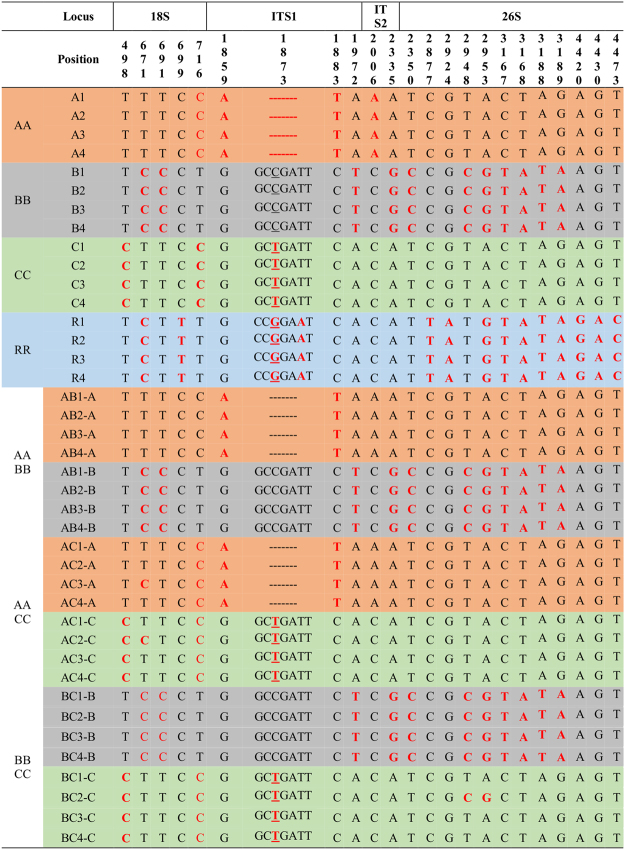
Summary of nucleotide variations based on 45SnrDNA sequences from 28 genotypes.

**Figure 4:**
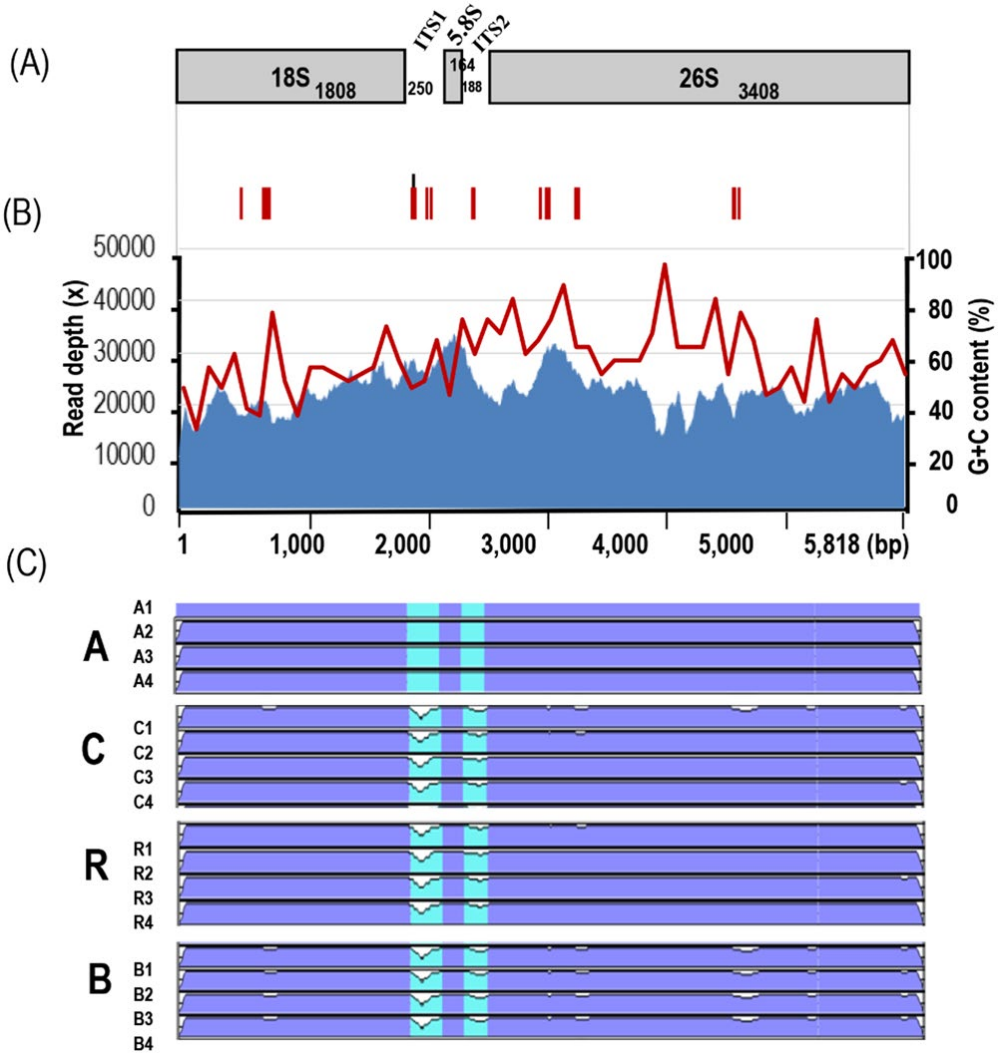
Structure and similarity analyses of 45SnrDNA sequences from 28 *Brassica* and *Raphanus* genotypes. (**A**) Complete structure and gene annotation of 45SrDNA sequences from the A1 genome. (**B**) Red and black arrowheads indicate the SNP and InDel variations, respectively. (**C**) Coverage of 45SnrDNA-based read mapping. Red lines indicate the proportion of G + C in the 4S 45SnrDNA. (**D**) Comparative analysis of similar and variable regions using mVISTA. Red arrowheads indicate inter-species variations.

The complete 45SnrDNA sequences of the four diploid species ranged from 5,816 to 5,831 bp (Table 1). Only one representative 45S was identified for each of the 16 diploid accessions of the A, B, C, and R genomes. By contrast, two different 45S sequences were identified for each of the tetraploid accessions. Therefore, 24 different 45SnrDNA sequences were identified for all 12 genotypes of the three allotetraploids (AB, AC, and BC genomes) (Table 1). Comparative analysis of the 40 types of 45SnrDNA sequences revealed 39 bp length variations in the 5,818 bp sequence (Figs 3, 4). Compared with the chloroplast genome, 45SnrDNA sequences were less diverse, with 22 SNPs and one InDel among 40 types of 45SnrDNA sequences from 28 genotypes. These variations were distributed among genic and intergenic regions.

Our analysis identified two types of 45SnrDNA (both parental) in three allotetraploids (AB, AC, and BC genomes). Each 45SnrDNA type in the three allotetraploids showed 100% sequence similarity with their corresponding parental diploid genome. For example, the A and B types of 45SnrDNA in the AB genome were 100% identical with those in the A and B genomes, respectively (Figs 3, 4). Read depth approach was used to estimate the copies of each 45SnrDNA type. Copy numbers differed among the allotetraploid sub-genomes, with 3,000–6,500 copies in the AB genome, 6,000–11,000 copies in the AC genome, and 2,200–2,900 copies in the BC genome. Copy numbers of each 45SnrDNA type in sub-genomes displayed a biased proportion up to 1.5-3-fold, with a higher proportion always occurring in the maternal ancestors of the AB, AC, and BC genomes (Table S5).

### Validation and utilization of species-specific variations

**Figure 5:**
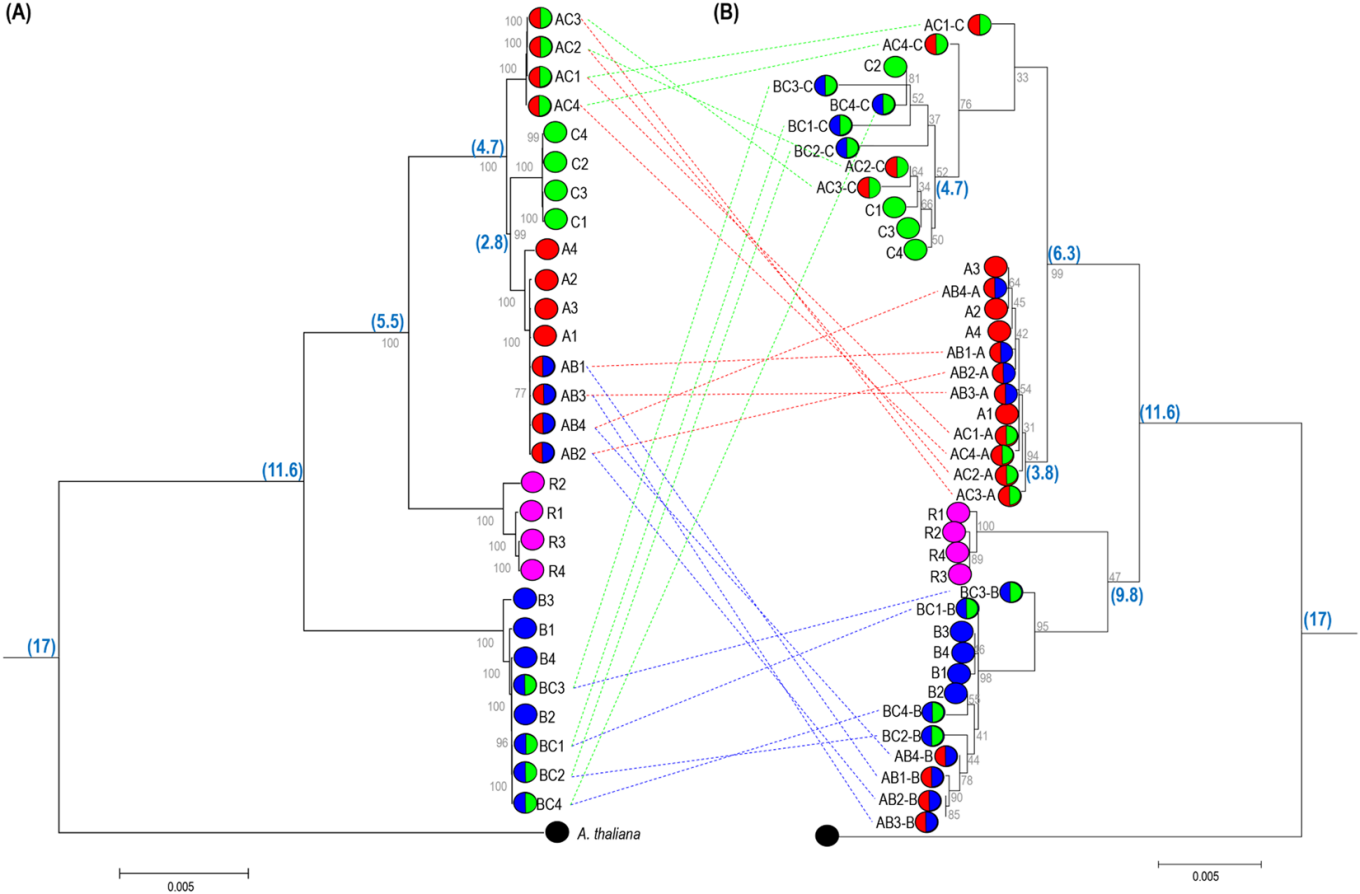
Phylogenetic relationships of the genus *Brassica* inferred from complete chloroplast (**A**) and 45SnrDNA (**B**) sequences of 28 *Brassica* and *Raphanus* genotypes. Tree was developed using MEGA7 with 1,000 bootstrap replications. The bootstrap values for clades are shown in corresponding branches of the tree. Taxon markers with single and double circles represent diploid and tetraploid genomes, respectively. The circled legend for 45SnrDNA and chloroplast corresponds to each species. Dotted line connects the corresponding allotetradiploid genomes of chloroplast and 45SnrDNA. Species divergence times were inferred from Bayesian analysis, and are shown at the side of the node in million years (my). *A*. *thaliana* was used as an outgroup.

Although chloroplast and 45SnrDNA sequences are highly conserved, our comparative analyses revealed a considerable number of variations (Fig. 5). There were more SNPs in chloroplast sequences than in 45SnrDNA sequences, with an average of 15 SNPs identified for every 1 kb of chloroplast genome, but only 3 for every 1 kb of 45SnrDNA. We tested the utility of this information on the diversity in chloroplast and 45SnrDNA sequences for identification and authentication of species or cultivars. We began with comprehensive analysis of the SNP and InDel variations in the chloroplast genome and 45SnrDNA to facilitate the development of barcode markers that enable the discrimination of each species. A total of 2,796 chloroplast variations were identified in 28 genotypes, and many of them were potential candidates for species-specific marker development (Tables S2, S3). We performed PCR analysis to validate the sequence polymorphism against several diversity-containing regions, and identified two InDel variations based on the chloroplast genome that could differentiate each diploid genome (A, B, C, and R genomes) (Figure S1). By contrast, only 23 variations (including 22 SNPs and one InDel) were identified based on 40 different 45SnrDNA sequences from 28 genotypes (Fig. 3). The 18S and ITS regions had relatively rich diversity and provide potential targets to differentiate the A, B, C, and R genomes by PCR analysis (Figure S2).

### Phylogenomic exploration of U’s triangle

Separate phylogenetic analyses based on chloroplast and 45SnrDNA sequences identified conserved genetic relationships and displayed essentially identical topologies among the *Brassica* species in U’s triangle. The high bootstrap values on the nodes support the reliability of the phylogenies produced based on both chloroplast and 45SnrDNA sequences (Fig. 5).

The chloroplast phylogenetic tree displayed five different clades, with clear discrimination between the four diploid genomes but an ambiguous clade in the AC genome (Figs 5A; S3). The four AC genomes were clustered with each other, but did not group with the parental A or C genomes. The other two allotetraploids (AB and BC) were clustered with one of their parental genomes; AB clustered with the A genome, and BC clustered with the B genome indicating that the A and B genomes were the maternal ancestors for the AB and BC genomes, respectively. However, the AC genome followed neither the A nor C genome as a source of maternal origin and showed an enigmatic relationship with its diploid parental genomes. Furthermore, we did not observe any reciprocal hybridization patterns in any of the three tetraploids.

The 45SnrDNA phylogenetic tree displayed clear classifications (Figs 5B, S4). The four diploid species diverged into four distinct clades, and each of the tetraploid genomes contained two independent 45SnrDNAs according to their corresponding ancestral maternal/paternal genomes. The AB genome harbored both A-type and B-type 45SnrDNA (AB-A and AB-B, respectively) with AB-A from the maternal genome and AB-B from the paternal. The BC and AC genomes also harbored two original subgenomic 45SnrDNA types; the B and C type in the BC genome (BC-B and BC-C, respectively), and the A and B type in the AB genome (AB-A and AB-B, respectively). Overall, the 45SnrDNA phylogenetic analysis of three alloteraploids revealed the expected parental association with the three diploids.

A phylogenetic tree generated by BEAST analysis showed similar topology as that generated by MEGA. The molecular dating based on chloroplast and 45SnrDNA sequences generated essentially similar divergence times (Figures S3, S4). Tree topologies with inferred speciation dates clearly identified three major divergence periods in both analyses (chloroplast and 45SnrDNA) of *Brassica*. The trees indicated that divergence and speciation in the B genome occurred 11 mya, followed by R genome divergence at 9 mya, and speciation of A and C genomes about 4.5 mya. The allotetraploids appear to have arisen from their diploid ancestors from 0.001 to 0.03 mya (Figure S5).

## Discussion

### Inter-species nucleotide diversity of chloroplast and 45SnrDNA

Chloroplast genomes are highly stable, have low mutation rates, and produce highly reliable phylogenetic trees that help elucidate plant evolutionary history^15,18,30,33^. Nuclear ribosomal DNAs can remain highly homozygous, whereas nuclear genomes are subject to cross-hybridization and cross-over during meiosis^32^. We produced a comprehensive diversity map for *Brassica* based on 28 complete chloroplast genomes and 40 types of complete 45SnrDNA sequences of the major species listed in the classical U’s triangle. The chloroplast and 45SnrDNA sequences exhibited highly conserved gene structures and gene orders at the inter-species and intra-species levels. However, considerable numbers of nucleotide variations were observed in both chloroplast and 45SnrDNA, which represent genus- and species-specific variations that can be developed for barcode markers and molecular breeding analysis. The B genome was highly diverged from the other genomes, suggesting prolonged, independent evolution. The estimated speciation time of the B genome is consistent with this result (Table 2). Phylogenetic analyses based on chloroplast genomes and 45SnrDNA sequences showed general agreement, with the B genome as a sister group to the A and C genomes and the derived allotetraploids following their corresponding progenitor genomes^6,34^. The chloroplast and 45SnrDNA sequences indicate that the R genome was closer to the A and C genomes then the B genome.

### Chloroplast genomes of seven species have different intra-species nucleotide diversity levels

Four genotypes for each species all showed different levels of divergent intra-species chloroplast genome polymorphism, although there were fewer intra-species polymorphisms than inter-species polymorphisms. The three allotetraploids rarely showed intra-species diversity (as expected) because those species were generated by allotetraploidization less than 0.05 mya^6,8^. Although four genotypes cannot represent the full diversity of each species, our results indicate that the C genome is less diverse than the maternal ancestor genome, the A and B genomes are moderately diverse, and the R genome is more diverse than the maternal ancestor genome. These results are consistent with a recent report of very low diversity in the C genome and relatively rich diversity in the A and B chloroplast genomes^23,35^. Similarly, rich variations were identified in the mitochondrial genome of *R*. *sativus*. Our previous work showed that dynamic mitochondrial genome rearrangements caused cytoplasmic male sterility and large variations among radish lines^36^ compared with the relatively conserved mitochondrial genome structures in *Brassica*^30,37^.

### Two types of 45SnrDNA are derived from two diploid ancestors in allotetraploids

Some polyploid plants maintain both parental ribosomal DNA genomes (5SnrDNA and 45SnrDNA) after allopolyploidization^38–41^. However, many allopolyploids express nucleolar dominance (ND), in which rRNA from one parent is transcriptionally silenced or recessively expressed^42,43^. ND is anticipated to have a significant role in chromatin modification and genome evolution^44,45^. Homogenization into one of two rDNA types also occurs via concerted evolution, mediated by rearrangements such as repeat loss, replacement, and recombination^46–49^.

There are few reports of the complete 45SnrDNA sequence in plant genomes. Here, we obtained the complete 45S rDNA transcription sequences for 28 accessions. We found only one highly homologous 45SnrDNA sequence in each accession of four diploids, but detected two types of 45SnrDNAs derived from the parental diploid ancestors in all three allotetraploids. Copy number analysis revealed that 45SnrDNA sequence bias toward the maternal genome occurred in the order of A > B > C genomes (Table S5), suggesting that there was genome-specific expansion of 45SnrDNA, which might be caused by sub-genome dominance^6^. However, further studies are required to address the consequences of rDNA copy number variation in allotetraploid *Brassica* (Table 1).

### Origin of the chloroplast genome in the AC genome accessions

Phylogenetic analyses conducted with only one or a few loci can misrepresent the derived phylogenic history, and complete information on genetic diversity is required for accurate analysis^50^. Unlike 45SnrDNA, chloroplast-based phylogenetic analysis indicates that the AC genome chloroplasts did not follow either of the parental nuclear genomes (A or C genome). Studies have been performed to clarify the genetic relationships of the major diploid and tetraploid *Brassica* species, but the origin of the chloroplast in the AC genome species is still unclear^15,20,21,51^. Initially, maternal parent of the AC genome was thought to be derived from the C genome due to their similarities in their chloroplast DNA restriction digest patterns; however, analysis with a wider range of accessions suggested that A genome was the maternal source^20^. Moreover, analysis with both chloroplast and nuclear markers suggested that the AC genome arose from several independent hybridization events including artificial introgression of A-genome^51^. A survey of the *rpo* locus revealed that >90% of 488 AC accessions displayed different genotypes than the parental accessions (A and C), but they were classified as an independent group with different origin^20^. Comparison with the recent findings of the *Brassica* chloroplast genome shows overall agreement, such as grouping based on species and the maternal and paternal origin of the allotetraploids^23^. Though there were two different A genome sources for the AC and AB genomes, we did not observe any divergence based on 45SnrDNA, suggesting that 45SnrDNA has been conserved in the *Brassica* genome^8^. Furthermore, chloroplast genomes from nine and seven different A and C genome morphotypes, respectively, formed a single cluster to confirm that the chloroplast genome and 45SnrDNA are stable even upon divergence of different sub-species and morphotypes^23^. In addition, sub-genome parallel selection played a crucial role in evolution of different morphotypes^52^.

Furthermore, A recent chloroplast genome survey of more diverse A genotypes revealed two different types of chloroplast genomes. The rapa-type1 chloroplast genome is generally found in all *B*. *rapa*, whereas the rapa-type2 is unique for some Italian Broccoletto genotypes of *B*. *rapa*^23^. Phylogenetic analysis indicated that the rapa-type2 clustered with the chloroplast of the AC genome, which explains why the Italian Broccoletto genotype is the donor for the most abundant AC chloroplast genome. The rapa-type2 chloroplast genome diverged 4.7 mya, which coincides with the currently known A and C genome divergence around 5.4 to 2.7 mya (Fig. 5). By contrast, analysis of the AC genome indicated that allotetraploidization occurred 7,500 years ago^6^. Both of these results indicate that the rapa-type2 chloroplast genome was maintained in the Italian Broccoletto genotype by geographical isolation or maternal dominance since 4.7 mya, and the Italian Broccoletto genome was utilized as the matenal parent to generate the AC genome 7,500 years ago. However, there are still questions about the evolution of the maternal genomes for the A and AC genomes. It is still not known how the rapa-type2 chloroplast genome became associated with the common maternal parent for most AC genomes, although the Italian Broccoletto genotype is not widespread in the A genome.

## Conclusion

This study analyzed the genetic relationships and diversity among *Brassica* species using chloroplast genome and 45SnrDNA sequences. Phylogenetic analysis revealed that the B genome diverged first in the *Brassica* clade, followed by R, A, and C, and with three allotetraploids forming during last 0.1 to 0.01 mya. We cataloged the complete variants in chloroplast and 45SrDNA sequences, which will serve as excellent resources for the development of barcode markers and species identification. Comparative genome analyses of species-specific variations would facilitate the study of genome evolution and morphological divergence of *Brassica*. The combined results of this study reveal comprehensive genetic relationships of U’s triangle species and provide insights into genome evolution in *Brassica*. The results of this study will be extensively applicable for species identification and evolutionary studies.

## Materials and Methods

### Plant materials and DNA sequencing

Seeds of four genotypes representing each A, B, C, R, AB, AC, and BC genome were obtained from the RDA Genebank Center, Suwon, South Korea. All plants were grown at 22 °C (day)/18 °C (night) with a 16 h light/8 h dark photoperiod at the RDA experimental farm, Suwon, South Korea, during the spring of 2014. High-quality total genomic DNA was isolated from young leaves using a modified CTAB method^53^. Whole-genome shotgun libraries were generated using the TrueSeq DNA PCR-Free Library Preparation kit (Illumina) according to the manufacturer’s instructions. Briefly, 5 ng of high-quality DNA from each accession was fragmented via sonication. Then, the fragments were end-repaired and A-tailed. Adapters were ligated, including the barcoding and multiplex identifier adapters, and the fragments were amplified with 10 PCR cycles. Finally, a paired-end (PE) library with inserts of 400−500 bp was generated. The library was sequenced with the MiSeq System (Illumina) at LabGenomics (www.labgenomics.co.kr, South Korea). Multiplex adapters were used to separate the 28 genotypes from the bulked raw reads, and the sequence reads were trimmed for adaptors and low quality and utilized for further analysis. All trimmed high quality sequences (NN3658-NN3685) for the 28 accessions were deposited into the National Agricultural Biotechnology Information Center (http://nabic.rda.go.kr) public database^54^ (Table S6).

### Assembly and annotation of chloroplast genome and 45SrDNA sequences

Complete chloroplast genome and 45SnrDNA sequences were simultaneously assembled for all 28 *Brassica* and *Raphanus* genotypes using the dnaLCW method^32^. The dnaLCW method is a fast and comparatively easy method that does not require a PCR based gap filling to assemble the chloroplast and rDNA sequences. With slight modification, dnaLCW also allows the characterization of the major repeats in the *Brassica* genome^55^. Briefly, high-quality Illumina paired-end reads were *denovo* assembled using the CLC genome assembler (ver. 4.06 beta, CLC Inc., Aarhus, Denmark) with autonomously controlled overlap size (200–500 bp). After gap closing, the resulting contigs were homology searched against the *Arabidopsis thaliana* chloroplast reference genome (GenBank accession: NC_000932) using mummer. Contigs related to the chloroplast genome were ordered according to the reference genome. Gaps and other errors such as false SNPs, copies of tandem repeats and homopolymer errors were corrected according to the dnaLCW approach^32^. Likewise, *Arabidopsis thaliana* 45SrDNA sequence (GenBank accession: X52322.1) was used as a reference to assemble the 45SnrDNA sequences of 28 genotypes. Due to the number of variations in intergenic spacer sequences (up to six types in *B*. *oleracea*), only the unique 45SnrDNA transcription units were assembled. We also identified both parental types of 45SnrDNA in an allotetraploid genome [i.e., *B*. *napus* (AC) genome] containing parental or sub-genomes of *B*. *rapa* (A) and *B*. *oleracea* (C), which were represented as AC-A and AC-C, respectively.

The chloroplast genomes of the 28 genotypes were annotated for protein-coding genes, transfer RNA (tRNA), and ribosomal RNA (rRNA) using DOGMA (https://dogma.ccbb.utexas.edu/)^56^. The accuracy of the start and stop codons and intron–exon boundaries were manually annotated based on previously annotated information from the close relative *A*. *thaliana*. The complete structure of tRNA genes was validated using tRNAscan-SE v1.2.1^57^. The systematic circular view of the chloroplast genome was created using OGDRAW and in-house customized perl script^58^. Comparative syntenic maps were generated using circos following the BlastZ annotation. A chloroplast-based browser was developed for systematic analysis of the chloroplast genomes of the 28 *Brassica* and *Raphanus* genotypes, which can be accessed at www.phyzen.co.kr/cpbrowser. The chloroplast browser also contains sequence and gene annotation information for all 28 genotypes. Similarly, 45SnrDNA genes (18S, 5.8S, and 26S) were annotated based on Blast analyses and reported reference units. The mvista tool was used to visualize comparative syntenic relationships with other genotypes^59^. Complete chloroplast genomes and 40 complete 45SnrDNA sequences from 28 genotypes were deposited in GenBank (Table 1).

### Structural variations and PCR analysis of chloroplast and 45SnrDNA

Extensive manual curation of chloroplast and 45SnrDNA revealed different kinds of non-redundant sequence variations (SV) such as SNPs, InDels, and copy number variations. Inter-species and intra-species structural variations were analyzed for chloroplast and 45SnrDNA sequences from 28 *Brassica* and *Raphanus* genotypes (Tables S2–S5). Putative SNPs and InDels were manually analyzed using the file aligned with MEGA7. Tandem repeats were identified using the Tandem repeats finder (TRF) tool. To detect highly reliable variations, all predicted variations were manually curated for both chloroplast and 45SnrDNA. Some of the randomly selected and highly informative variations were validated by PCR analysis.

To validate the polymorphic regions of chloroplast and 45SnrDNA sequences, specific primers were developed for high-quality structural variations such as SNPs and InDels (Table S7). DNA templates from 28 genotypes were used for target analysis. Each PCR reaction contained 10 ng template DNA, 10 pM primers, 0.5 µM dNTPs, 2 units of Taq polymerase (TAKARA, Japan), and the final volume brought to 20 µl with sterile distilled water. The PCR reactions were 10 min at 95 °C; followed by 36 cycles of 30S at 94 °C, 30S at 55−62 °C, and 30S at 72 °C; with a final extension at 72 °C for 5 min. Amplified fragments were checked with 2% agarose gel electrophoresis to estimate the product size.

### Phylogenetic analysis and divergence estimation based on chloroplast genomes and 45SnrDNA sequences

Complete chloroplast genomes and 45SnrDNA sequences were independently explored for phylogenetic and divergence analysis. Chloroplast sequences of 28 *Brassica* and *Raphanus* genotypes were aligned with a previously reported *Brassica* chloroplast sequence using MAFFT (http://mafft.cbrc.jp/alignment/software/). Phylogenetic trees were constructed in MEGA7 using the neighbor-joining iterative model with 1,000 bootstrap replications^60^. Phylogenetic analysis was performed for 40 types of 45SnrDNA sequences based on 28 genotypes. *A*. *thaliana* chloroplast and 45SnrDNA sequences were used as an outgroup for the phylogenetic analysis. The reference chloroplast sequence with its annotation of *A*. *thaliana*, *B*. *rapa*, *B*. *oleracea*, *B*. *nigra*, *B*. *juncea*, *B*. *carinata*, and *Raphanus sativus* was obtained from GenBank.

Chloroplast and 45SnrDNA sequences from 28 genotypes were subjected to tree topology analysis and divergence time estimation using Bayesian methods implemented in BEAST (http://beast.bio.ed.ac.uk/)^61^. The BEAST program assumes auto-correlation, and is widely used to estimate the uncertainty of divergence dates and branch lengths, to estimate divergence using known speciation dates, and to accommodate the branching rate. The GTR + I + G substitution model was used to construct the tree topology and divergence time. We used an uncorrelated lognormal relaxed clock model to perform 10,000,000 generations of Markov chain Monte Carlo (MCMC) analysis with sampling every 1,000 generations. A Yule tree prior were used to generate the random starting tree. Tracer v. 1.6 was used to obtain the BEAST run after discarding 10% of the generations as burn-in. The remaining BEAST runs were used for the posterior possibilities. The divergence time was estimated using Tree annotator. *A*. *thaliana* was constrained as the outgroup, and the age of divergence between *A*. *thaliana* and *Brassica* lineages was constrained by a normal distribution with a mean of 17 million years (my) and standard deviation of 2 my^9^.

### Availability of data and materials

All data generated or analysed during this study were obtained from the accession numbers provide at Tables 1 and S6.

## Electronic supplementary material

Supplementary Figures S1-S5

Supplementary Tables S1-S7
