# Supplementary material for: Re-exploration of U’s Triangle Brassica Species Based on Chloroplast Genomes and 45S nrDNA Sequences
Source: Sci Rep. 2018 May 9;8:7353. doi: 10.1038/s41598-018-25585-4 (PMC5943242; doi:10.1038/s41598-018-25585-4)
Supplement: Supplementary file 1 — Supplementary Figures S1-S5 [file 41598_2018_25585_MOESM1_ESM.pdf]

Re-exploration of U's Triangle *Brassica* Species Based on Chloroplast Genomes and 45S nrDNA Sequences

Chang-Kug Kim[1+], Young-Joo Seol [2+], Sampath Perumal[3,4+], Jonghoon Lee[3,5], Nomar Espinosa Waminal[3], Sang-Choon Lee[3], Murukarthick Jayakodi[3], Seungwoo Jin[3], Beom-Soon Choi[6], Yeisoo Yu[6], Ho-Cheol Ko[7], Ji-Weon Choi[8], Kyoung-Yul Ryu[2], Seong-Han Sohn[1,] Isobel Parkin[4] and Tae-Jin Yang[3,9*]

The following Supporting Information is available for this article:

Figure S1. Species-specific InDel variations and validation for *Brassica* and relatives based on chloroplast genomes. (A) Karyogram showing specific intergenic InDel variations for the A+B, C, and R genomes. Intragenomic variations were observed for the R genome. (B) Gel validation of InDel variations in 28 genotypes with grouping based on polymorphisms. Red triangles mark intragenomic variations in the R genome. (C) Karyogram showing specific genic InDel variations for the A+C, B, and R genomes. (D) Gel validation of InDel variations in 28 genotypes with grouping based on polymorphisms.

Figure S2. Potential regions for differentiation of each diploid genome based on 45SnrRNA variations. Boxed letters indicate species-specific SNPs; black arrowheads represent InDels.

Figure S3. Phylogenetic relationships and molecular dating of the genus *Brassica* based on complete chloroplast genomes. (A) Neighbor-joining tree inferred from complete chloroplast genomes from 28 accessions. Tree was developed using MEGA7 with 1,000 bootstrap replications. (B) Chronogram of *Brassica* genus inferred from Bayesian analysis as implemented in the BEAST program based on complete chloroplast genomes. Divergence times of species on the right of the node are in million years (my). Double arrowhead indicates the estimated divergence of the three allotetraploid (AB, AC, and BC) genomes.

Figure S4. Phylogenetic relationships and molecular dating of the genus *Brassica* based on nrDNA sequences. (A) Neighbor-joining tree inferred from complete 45SnrDNA sequences from 28 genotypes. Tree was developed using MEGA7 with 1,000 bootstrap replications. The bootstrap values for clades are shown in corresponding branches of the tree. (B) Divergence times of species on the right of the node are in million years (my). Double arrowhead indicates the estimated divergence of the three allotetraploid (AB, AC, and BC) genomes.

Figure S5. Evolutionary time line of U's triangle *Brassica* species based on this study and previous reports

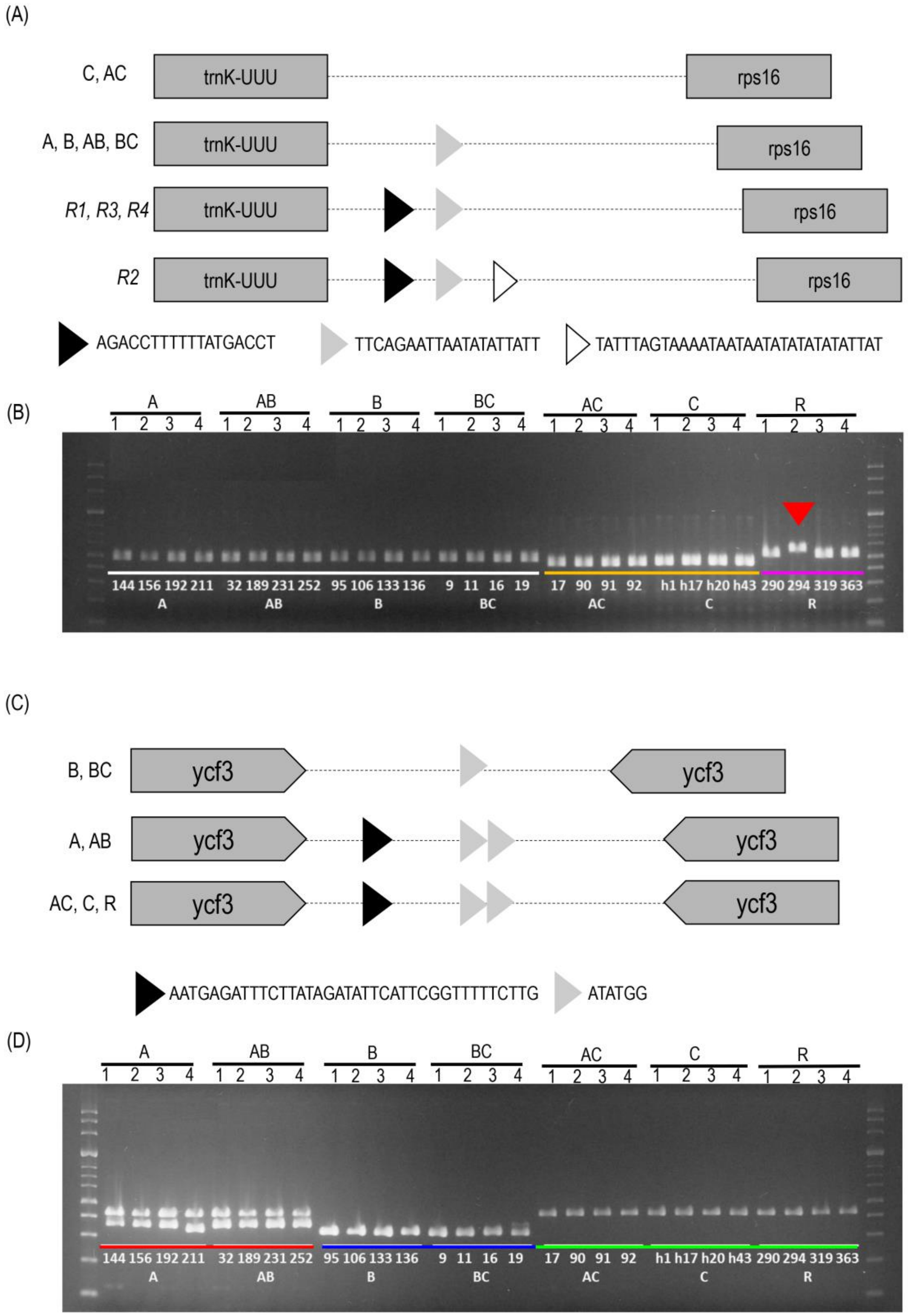
(A)
C, AC
A, B, AB, BC
R1, R3, R4
R2
trnK-UUU
rps16
AGACCTTTTTTATGACCT
TTCAGAATTAATATATTATT
TATTTAGTAAAATAATAATATATATATATTAT
(B)
A
AB
B
BC
AC
C
R
1 2 3 4
144 156 192 211
32 189 231 252
95 106 133 136
9 11 16 19
17 90 91 92
h1 h17 h20 h43
290 294 319 363
(C)
B, BC
A, AB
AC, C, R
ycf3
AATGAGATTTCTTATAGATATTCATTCGGTTTTTCTTG
ATATGG
(D)
A
AB
B
BC
AC
C
R
1 2 3 4
144 156 192 211
32 189 231 252
95 106 133 136
9 11 16 19
17 90 91 92
h1 h17 h20 h43
290 294 319 363

Figure S1.

.

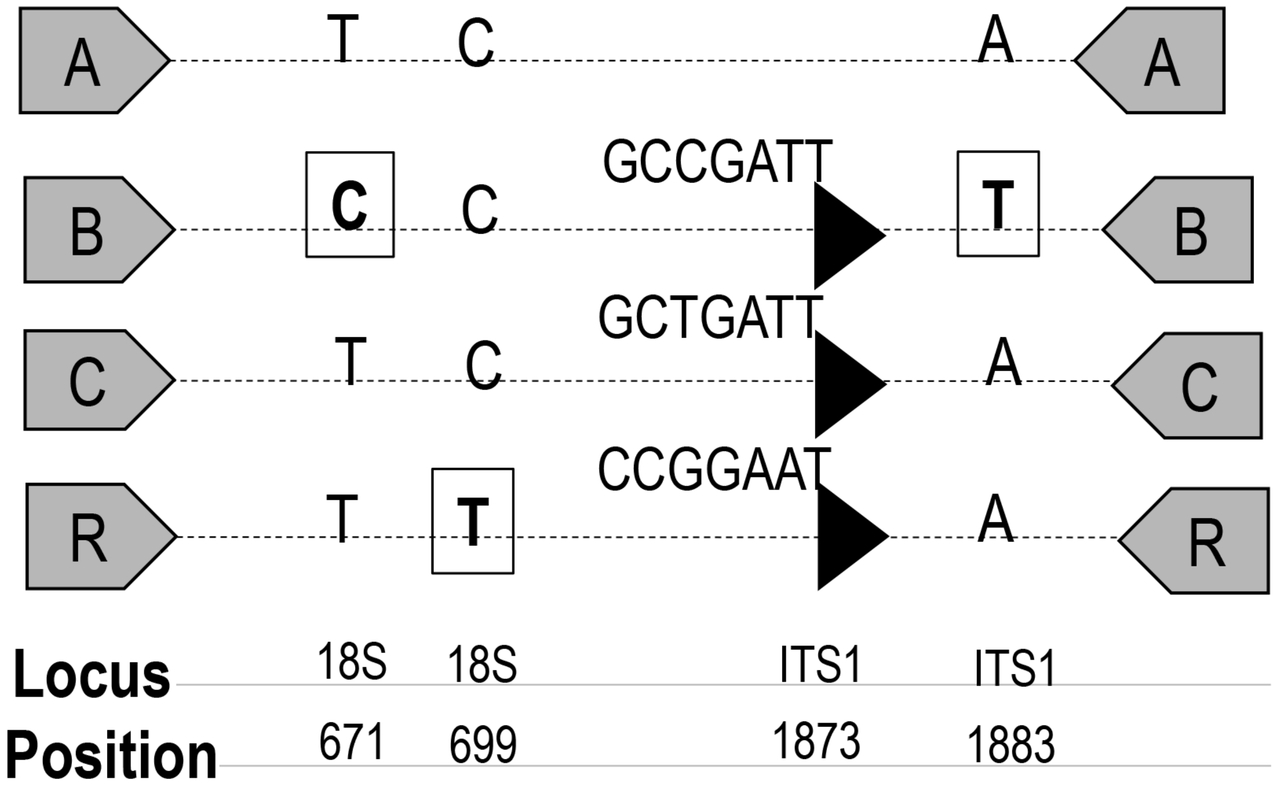
A
T
C
A
A
B
C
C
GCCGATT
T
B
C
GCTGATT
T
C
A
C
R
T
T
CCGGAAT
A
R
Locus
18S
18S
ITS1
ITS1
Position
671
699
1873
1883

Figure S2

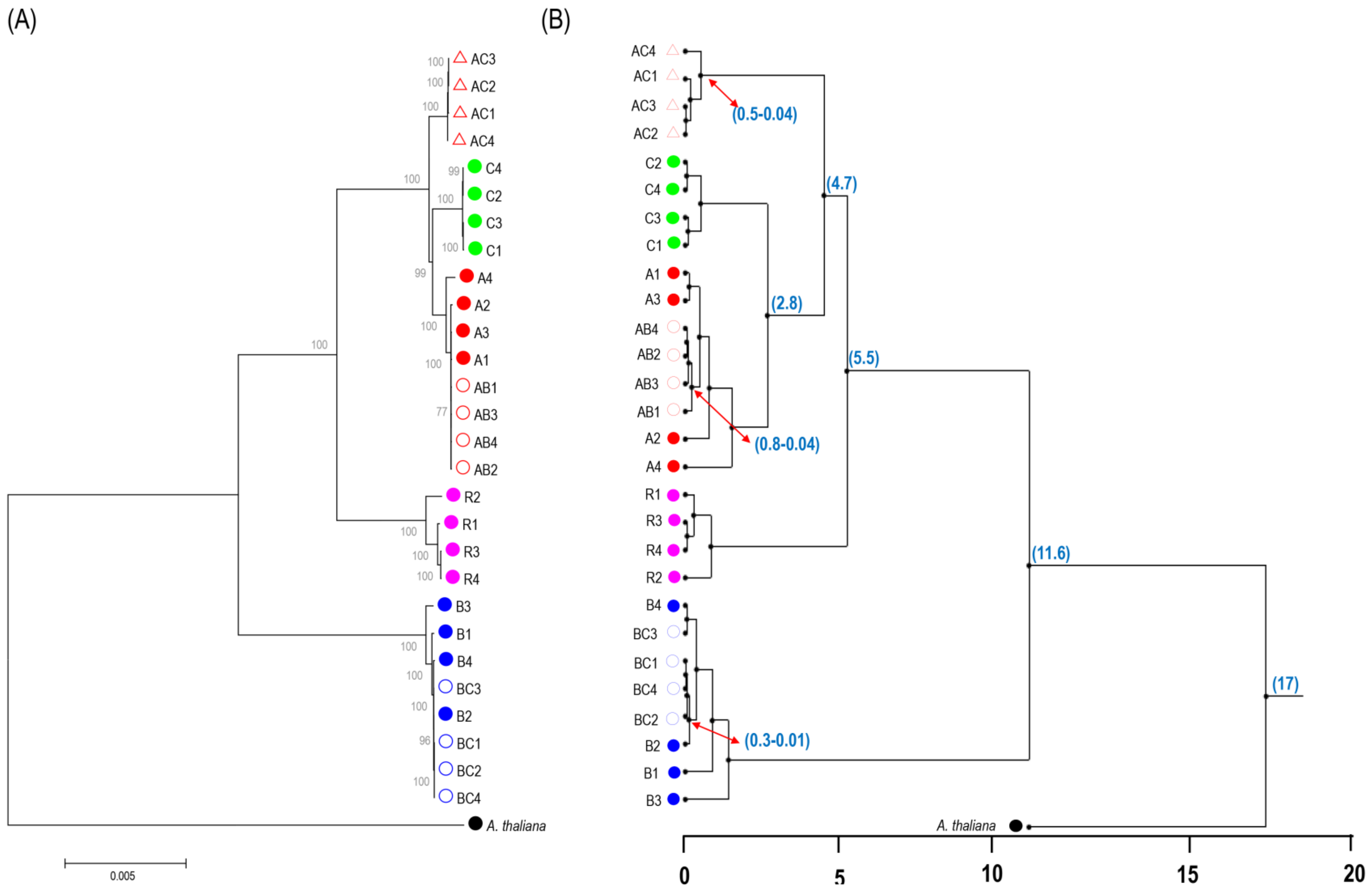
(A)
AC3
AC2
AC1
AC4
C4
C2
C3
C1
A4
A2
A3
A1
AB1
AB3
AB4
AB2
R2
R1
R3
R4
B3
B1
B4
BC3
B2
BC1
BC2
BC4
A. thaliana
0.005
(B)
AC4
AC1
AC3
AC2
(0.5-0.04)
C2
C4
C3
C1
(4.7)
A1
A3
(2.8)
AB4
AB2
(5.5)
AB3
AB1
A2
(0.8-0.04)
A4
R1
R3
R4
R2
(11.6)
B4
BC3
BC1
(17)
BC4
BC2
(0.3-0.01)
B2
B1
B3
A. thaliana
0
5
10
15
20

Figure S3

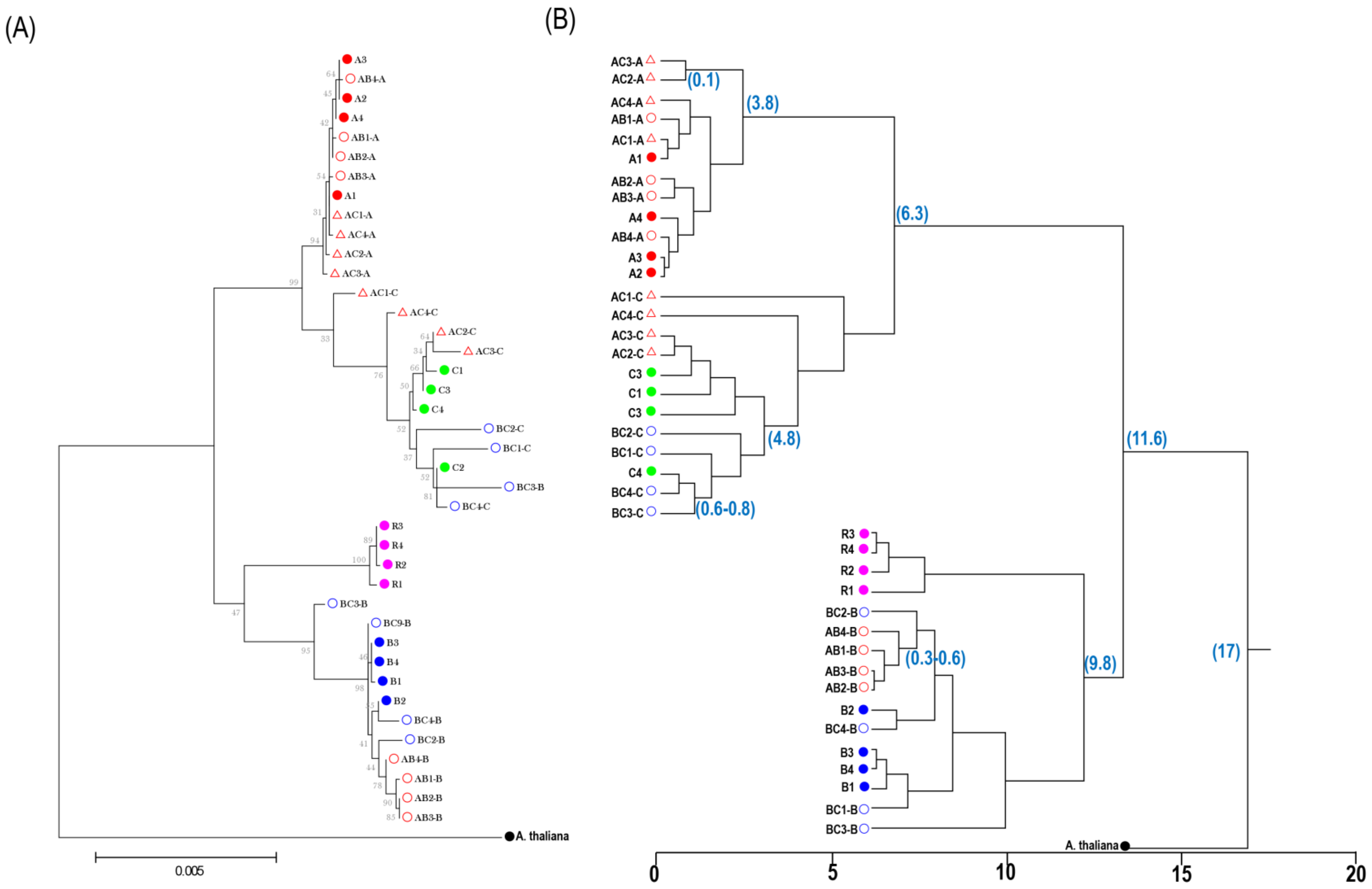
(A)
A3
AB4-A
A2
A4
AB1-A
AB2-A
AB3-A
A1
AC1-A
AC4-A
AC2-A
AC3-A
AC1-C
AC4-C
AC2-C
AC3-C
C1
C3
C4
BC2-C
BC1-C
C2
BC3-B
BC4-C
R3
R4
R2
R1
BC3-B
BC9-B
B3
B4
B1
B2
BC4-B
BC2-B
AB4-B
AB1-B
AB2-B
AB3-B
A. thaliana
0.005
(B)
AC3-A
AC2-A
AC4-A
AB1-A
AC1-A
A1
AB2-A
AB3-A
A4
AB4-A
A3
A2
AC1-C
AC4-C
AC3-C
AC2-C
C3
C1
C3
BC2-C
BC1-C
C4
BC4-C
BC3-C
(0.1)
(3.8)
(6.3)
(4.8)
(0.6-0.8)
(11.6)
R3
R4
R2
R1
BC2-B
AB4-B
AB1-B
AB3-B
AB2-B
B2
BC4-B
B3
B4
B1
BC1-B
BC3-B
(0.3-0.6)
(9.8)
(17)
A. thaliana
0
5
10
15
20

Figure S4

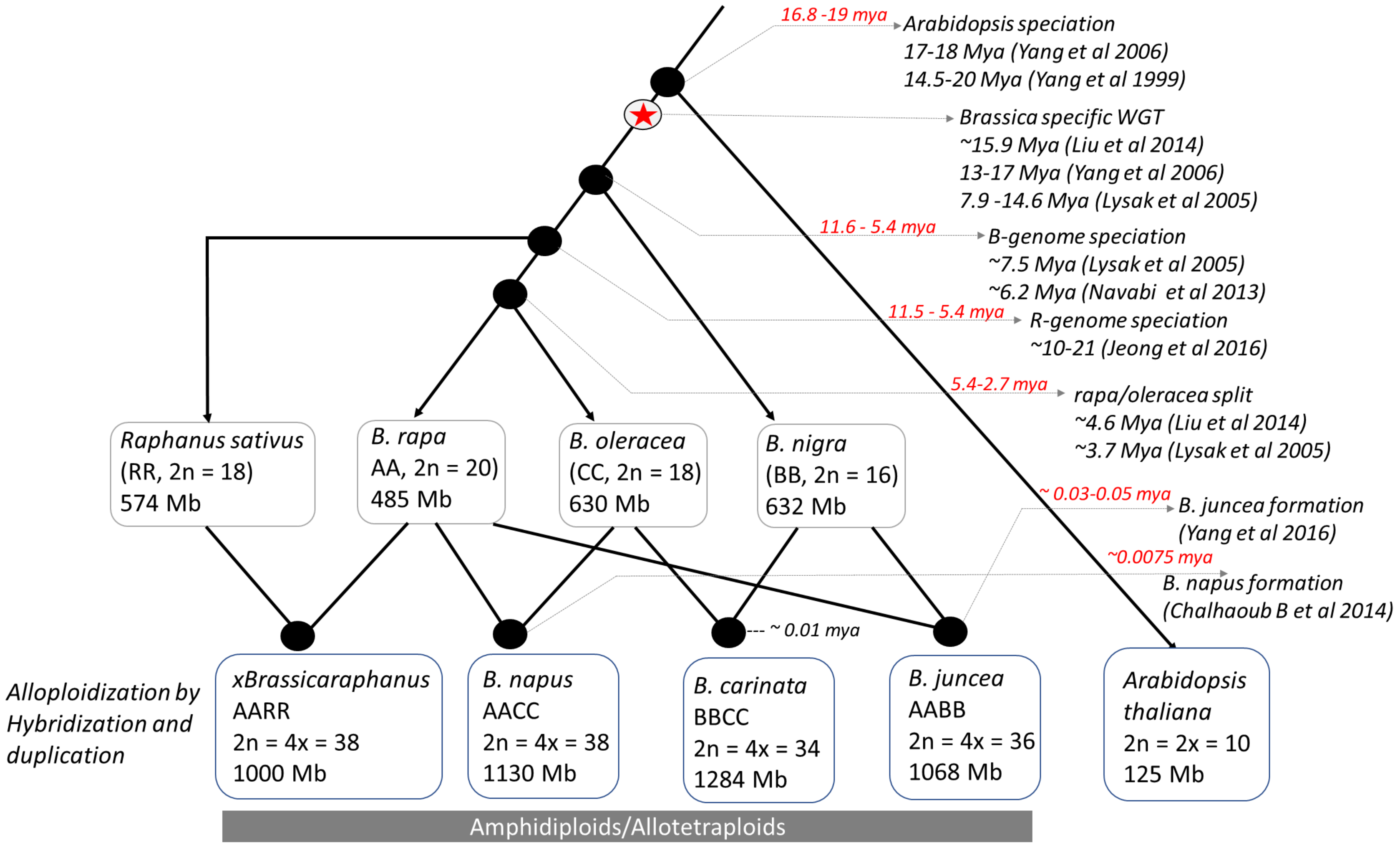
16.8 -19 mya
Arabidopsis speciation
17-18 Mya (Yang et al 2006)
14.5-20 Mya (Yang et al 1999)
Brassica specific WGT
~15.9 Mya (Liu et al 2014)
13-17 Mya (Yang et al 2006)
7.9 -14.6 Mya (Lysak et al 2005)
11.6 - 5.4 mya
B-genome speciation
~7.5 Mya (Lysak et al 2005)
~6.2 Mya (Navabi et al 2013)
11.5 - 5.4 mya
R-genome speciation
~10-21 (Jeong et al 2016)
5.4-2.7 mya
rapa/oleracea split
~4.6 Mya (Liu et al 2014)
~3.7 Mya (Lysak et al 2005)
Raphanus sativus
(RR, 2n = 18)
574 Mb
B. rapa
AA, 2n = 20)
485 Mb
B. oleracea
(CC, 2n = 18)
630 Mb
B. nigra
(BB, 2n = 16)
632 Mb
~ 0.03-0.05 mya
B. juncea formation
(Yang et al 2016)
~0.0075 mya
B. napus formation
(Chalhaoub B et al 2014)
--- ~ 0.01 mya
Alloploidization by
Hybridization and
duplication
xBrassicaraphanus
AARR
2n = 4x = 38
1000 Mb
B. napus
AACC
2n = 4x = 38
1130 Mb
B. carinata
BBCC
2n = 4x = 34
1284 Mb
B. juncea
AABB
2n = 4x = 36
1068 Mb
Arabidopsis
thaliana
2n = 2x = 10
125 Mb
Amphidiploids/Allotetraploids

Figure S5
